# Changes in health care inequity in Brazil between 2008 and 2013

**DOI:** 10.1186/s12939-016-0431-8

**Published:** 2016-11-17

**Authors:** Pricila Mullachery, Diana Silver, James Macinko

**Affiliations:** 1 College of Global Public Health, New York University, 411 Lafayette Street, 5th floor, New York, NY 10003 USA; 2 Departments of Health Policy and Management and Community Health Sciences, University of California Los Angeles Fielding School of Public Health, 650 Charles E. Young Dr. South, Room 31-235B, Center for Health Sciences, Los Angeles, CA 90095-1772 USA

**Keywords:** Brazil, Health care inequity, Health care utilization, Horizontal Equity Index

## Abstract

**Background:**

Brazil has made progress towards a more equitable distribution of health care, but gains may be threatened by economic instability resulting from the 2008 global financial crisis. This study measured predictors of health care utilization and changes in horizontal inequity between 2008 and 2013.

**Method:**

Data were from two nationally representative surveys that measured a variety of sociodemographic, health behaviors and health care indicators. We used Poisson regression models to estimate adjusted prevalence ratios and the Horizontal Equity Index (HEI) standardized by health needs to measure inequity in the utilization of doctor and dentist visits, hospitalizations and reporting of a usual source of care (USC) for those 18 and older. To estimate the HEI, we ranked the population from the poorest to the richest using a wealth index. We also decomposed the HEI into its different components and assessed changes from 2008 to 2013.

**Results:**

The population proportion with doctor and dentist visits in the past year and a USC increased between 2008 and 2013, while hospitalizations declined. In 2013, pro-rich inequity in doctor visits increased significantly while the distribution of hospitalizations shifted from pro-rich in 2008 to neutral in 2013. Dentist visits were highly pro-rich and USC was slightly pro-rich; the distribution of dentist visits and USC did not change over time. Health need was a strong predictor of health care utilization regardless of the type of coverage (public or private). Education, wealth, and private health plans were associated with the pro-rich orientation of doctor and dentist visits. Private health plans contributed to the pro-rich orientation of all outcomes, while the Family Health Strategy contributed to the pro-poor orientation of all outcomes.

**Conclusion:**

The results of this study support the claim that Brazil’s population continued to see absolute gains in access to care despite recent economic crises. However, gains in equity have slowed and may even decline if investments are not maintained as the country enters deeper financial and political crises.

**Electronic supplementary material:**

The online version of this article (doi:10.1186/s12939-016-0431-8) contains supplementary material, which is available to authorized users.

## Background

Brazil is a middle-income country with high income inequality and large regional disparities in health outcomes, infrastructure, and availability of public services [1–5]. Equity is a core guiding principle of the Brazilian national health system [6], the *Sistema Único de Saúde* (SUS) and in the past twenty years the country has invested significantly in policies to mitigate disparities in health and health care [7, 8]. In 2012, overall health spending in Brazil was close to the OECD average (about 9 %) [9] but with a markedly lower government share. Private spending (premiums and out-of-pocket payments) represented over 50 % of healthcare expenditures and about 26 % of Brazilians had a private health plan in 2013 [10].

In the SUS, primary health care is largely provided via a network of public facilities with multidisciplinary health teams that attend families living in geographically defined areas. This model of care, known as the Family Health Strategy (FHS), is based on core primary health care principles such as first-contact access and provision of comprehensive, continuous and coordinated care. Services offered by most FHS teams include doctor consultations, dental care, preventive exams, and home visits [11]. Over the past fifteen years, the expansion of the FHS in the poorest regions of the country improved access to care among the most vulnerable populations [2]. In addition to the FHS, health care access was expanded with the construction of new ambulatory care facilities and the improvement of the existing public services available to everyone free of charge [12].

A substantive literature has shown that, since the 1990s, healthcare utilization increased and unmet healthcare needs decreased in Brazil concurrent with the development of the SUS [7, 8, 13, 14]. However, this literature also points to an increase in perceived barriers to access, often related to changes in people’s expectations and overcrowding of facilities due to a growing demand for services. In terms of equity, studies have shown that individuals with less income and education and without formal employment are more likely to face barriers to care, and present lower levels of utilization, after controlling for age and health status, than their counterparts [15–17].

However, overall, disparities in health care utilization have declined over time. The Horizontal Equity Index (HEI) (used to measure inequity in health care utilization) employs Lorenz curves to plot the cumulative proportion of services utilized over the population ordered from the poorest to the richest, with utilization being standardized by variables indicative of health needs [18]. The HEI thus allows for the estimation of inequity, or the inequality that remains after health needs are taken into account. Two previous studies that assessed horizontal inequity in health care in Brazil pointed to a decline in pro-rich inequity--meaning that use of services became less concentrated among the richest from 1998 to 2008 [19, 20].

In a broader context, over the past fifteen years, Brazil has undergone substantial social and economic transformations with declines in poverty and improvements in living conditions [21]. However, recent economic instability resulting from the 2008 global financial crisis has raised concerns about increasing barriers to care among the poorest. Evidence from OECD countries suggests a pattern of decline in the growth of public health expenditures and reduced service availability following major economic crises [22]. Vulnerable populations are often the hardest hit, being more likely to face income reduction, job loss and increased barriers to health care [23, 24]. While Brazilian governmental measures taken after the first shock in 2008 seem to have mitigated the effect of the crises in the short term [25], after 2011 with the continuous decline in commodity prices, Brazil’s economy began to contract [26].

To our knowledge, there are no studies assessing changes in ﻿health care inequity﻿ during the recent period of economic instability in Brazil. This study fills that gap by identifying predictors of health care utilization and measuring changes in horizontal inequity between 2008 and 2013.

## Methods

### Data sources

We use data from two nationally representative surveys that are publically available. The first, the *Pesquisa Nacional por Amostra de Domicílios* (PNAD) conducted in 2008, and the second, the *Pesquisa Nacional de Saúde* or National Health Survey conducted in 2013. Both surveys had complex probabilistic sample design. The PNAD was designed in three stages: municipality, census tract, and households. Municipalities and census tracts were selected with probability proportional to the size of the resident population and number of households, respectively. The households in each census tract were randomly selected from the national registry of addresses and in each household a proxy respondent answered questions regarding all household members. A total of 150,591 households were included in the PNAD﻿ with 391,868 residents [27], among which 271,677 were 18 and older. The PNS also had three stages: census tract, household, and resident 18 and older. Random selection was used in all stages of the PNS, which included 64,348 households totaling 205,000 residents. Among those, 60,202 individuals were selected to respond to an individual questionnaire. For each survey, final individual weights were calculated considering the inverse probability of being selected in different stages. All respondents participated via face-to-face interview. Response rates for both surveys were close to 80 % [8, 27]. A detailed description of the PNAD and PNS sampling strategies can be found elsewhere [28].

PNAD and PNS included similar blocks of questions regarding demographic and socioeconomic background, health status, and health care utilization patterns. However, in 2013, the questionnaire had two parts, the first directed to all household members and answered by a proxy resident, and the second answered by one randomly selected adult resident. Some questions were only available in the second part (applied to the 60,202 selected residents). In order to include the largest number of predictors and to maintain the three-stage sample design in both years, the household members not selected to respond to the second part of the questionnaire were dropped from the PNS 2013 sample used in this study.

### Measures

#### Measure of health care utilization (outcomes)

We used four commonly assessed measures of health care utilization aiming to capture different patterns of inequity across types of services [29]; doctor visits, dentist visits, hospital admission and reporting of usual source of care, this last outcome used as proxy for care continuity [30]. Dichotomous measures were derived from the questions: “Have you consulted with a doctor in the past 12 months?”, “Have you consulted with a dentist in the past 12 months?”, “Have you been admitted to a hospital in the past 12 months?”, and “Do you usually use the same healthcare service or doctor when you need care?”

### Independent variables

#### Measure of socioeconomic position

Socioeconomic position was determined by an index composed of seventeen variables of housing characteristics (number of rooms and bathrooms, sewage and garbage disposal, cooking method, running water, electricity) and consumer durable goods (e.g. cell phone, TV, fridge, computer, washer, car, among others). We used Principal Component Analysis (PCA) to reduce the number of observed variables to one principal component. The wealth index was generated separately for each year by extracting the first component of the PCA model. Cronbach's alpha was 0.85 and 0.86 in 2008 and 2013 respectively, indicating good internal consistency of the variables included in the index.

#### Covariates of health care utilization

Covariates of health care utilization were determined according to the literature [31] and included sex, age, race, place of residency (rural v. urban and region of the country), literacy (whether the person can read and write), level of schooling (less than primary, primary complete, secondary complete, and college or more), indicators of health status (self-rated health, reporting of activities interrupted due to health issues, reporting of selected chronic diseases and comorbidity), and type of healthcare coverage.

To simplify analyses some covariates were dichotomized -- race was dichotomized to white vs. non-white and wealth was dichotomized to poor (bottom two quintiles) vs. non-poor (top three quintiles of the wealth index). To measure health needs we applied factor analysis to variables indicative of self-reported health status, activities interrupted due to health problems, self-reported chronic diseases, and comorbidity. The latent variable resulting from the factor analysis was then categorized into low need (bottom 50 %), moderate need (middle 25 %) and high need (top 25 %). Healthcare coverage was categorized as: “Private plan”, indicating people who reported having a private health plan; “FHS”, indicating people who reported being enrolled in the Family Health Strategy and excluding those who also reported a private plan; and “UBS/SUS”, indicating those who did not report a private plan or FHS but are free to use public health clinics (basic healthcare units or UBS) or any other facility in the SUS network.

### Analytic strategy

We used a variety of methods in this study. First, we combined data from both time periods taking steps to maintain the integrity of the survey design regarding the primary sampling units and strata defined in each year. All variables were recoded to assure consistency and then appended to create a joint dataset. We compared weighted estimates of demographic characteristics and outcome prevalence rates from our combined dataset with those resulting from the PNAD and PNS separately and confirmed that the combined dataset was pristine. Then, we used Poisson regression models to measure change in utilization between 2008 and 2013 and to estimate adjusted prevalence ratios for predictors of each outcome. We tested for interactions between poverty and type of coverage, and health needs and coverage to examine whether these factors affected health care utilization differently. The interaction poverty-coverage was significant for all outcomes. The interaction health needs-coverage was significant for all outcomes except dentist visit. Only these significant interactions were kept in the final models.

Second, we used the Horizontal Equity Index (HEI) to assess inequity in the distribution of health care services. We used the indirect method to standardize the HEI by need [29], with need variables including sex/age categories (11 dummy variables representing men and women 18–24, 25–34, 35–44, 45–54, 55–64, 65 and older, with men 18–24 used as the reference), and health needs (moderate and high health needs, with low needs used as the reference). The HEI normally varies between−1 and 1, with positive values representing pro-rich distribution and negative values representing pro-poor distribution. However, according to Wagstaff (2005) [32], when we deal with binary outcomes, the bounds of measures such as the HEI change according to the prevalence of the outcome. In this case, the HEI lower and upper limits would be equal to P-1 and 1-P, respectively, where P is the prevalence. In our study, prevalence-adjusted HEIs were calculated as a percentage of the total inequity possible, represented by the new lower (P-1) or upper bounds (1-P). This adjustment is especially important when the prevalence of the outcome changes over time since the percentage represented by the HEI value will also change as a result of the new bounds.

Third, we used a decomposition method to measure the contribution of need (age, sex and health needs) and non-need factors (all the other covariates) to the estimated HEI [33]. The results of the decomposition analysis produced estimates of the size and type of the contribution (pro-rich or pro-poor) for each predictor [34]. HEI estimation and decomposition were performed for 2008 and 2013 separately. All analysis included probability weights and adjustment for the survey design. We used the software Stata version 13 [35] to generate descriptive tables and Poisson models, and the software ADePT [36] to estimate the HEIs and factors’ contribution. ADePT output included the standard errors for the HEI, which were used to construct 95 % confidence intervals.

## Results

Table 1 shows the sample characteristics. The age distribution changed significantly between 2008 and 2013, with declines in the groups below the age of 45 and increases in the groups 45 and older. Roughly half of the respondents self-classified as white with a small but significant decline in 2013. In 2013, 8 % of the population reported not knowing how to read or write -- a statistically significantly decline from 2008. Education attainment also improved, with increases in the percentage of people who completed high school and college. The proportion of residents in rural areas dropped slightly over the period. Reporting fair or poor health increased significantly from 2008 (﻿29 ﻿%) to 2013 (32﻿ %), as did reporting of chronic diseases (from 32 to 35 %). However, reporting of activities interrupted due to health issues decreased slightly. Less than one-sixth reported two or more chronic diseases in both years. Enrollment in the Family Health Strategy was reported by nearly half of the respondents in 2008, increasing to 55 % in 2013, while private health plan coverage remained the same (26 %). Utilization of doctor visits in the past year (70 % v. 75 %), dentist visits in the past year (39 % v. 44 %), and usual source of care (73 to 77 %) also increased over time. Hospitalizations in the past year declined slightly. A comparison of the sample characteristics and prevalence rates between separate datasets and our combined dataset showed identical point estimates (Additional file 1).

**Table 1:** Sample characteristics, Brazil, PNAD 2008 and PNS 2013

Characteristics	2008		2013	
	%	95 % CI	%	95 % CI
Social and demographic				
Women	52.32	[52.15,52.49]	52.9	[52.10,53.69]
Age				
18–24	17.51	[17.32,17.70]	15.93**	[15.32,16.55]
25–34	23.23	[22.99,23.47]	21.63**	[21.00,22.26]
35–44	20.33	[20.12,20.54]	19.19**	[18.60,19.80]
45–54	16.87	[16.68,17.06]	17.5*	[16.92,18.09]
55–64	11.11	[10.94,11.28]	13.46**	[12.94,14.00]
65 +	10.95	[10.76,11.14]	12.29**	[11.76,12.85]
Race/ethnicity				
White	49.98	[49.14,50.83]	47.46**	[46.44,48.49]
Non-white	50.02	[49.17,50.86]	52.54**	[51.51,53.56]
Rural residence	15.37	[14.43,16.35]	13.79*	[13.31,14.29]
Region				
North	7.35	[6.43,8.39]	7.44	[7.01,7.89]
Northeast	26.42	[24.67,28.24]	26.62	[25.42,27.85]
Southeast	44.14	[42.69,45.60]	43.79	[42.32,45.28]
South	14.82	[14.29,15.36]	14.78	[13.89,15.72]
Center	7.27	[7.00,7.55]	7.36	[6.96,7.79]
Illiterate	10.6	[10.13,11.14]	8.48*	[8.03,8.95]
Education level				
Less than primary education	45.88	[45.34,46.42]	38.93**	[38.02,39.85]
Primary complete	16.42	[16.19,16.65]	15.53*	[14.95,16.12]
Secondary complete	29.01	[28.63,29.40]	32.80**	[32.05,33.57]
College complete or more	8.69	[8.39,8.99]	12.74**	[11.99,13.52]
Health Status				
Fair/poor self-rated health^a^	28.7	[28.34,29.05]	32.24**	[31.48,33.00]
Activity interrupted due to health	8.93	[8.73,9.13]	8.06**	[7.65,8.49]
At least one chronic disease^b^	31.91	[31.60,32.23]	35.34**	[34.60,36.08]
Two or more chronic diseases	12.22	[12.01,12.43]	12.71	[12.20,13.23]
Health care coverage				
Enrolled in the Family Health	48.79	[47.78,50.10]	54.61**	[53.18,56.03]
Enrolled in private plan	26.16	[25.64,26.69]	26.40	[25.47,27.34]
Health care utilization				
Doctor visit	69.81	[69.44,70.18]	74.20**	[73.43,74.96]
Dentist visit	38.97	[38.53,39.43]	44.43**	[43.58,45.29]
Hospitalization^c^	6.66	[6.51,6.81]	5.73**	[5.39,6.08]
Usual Source of Care	72.89	[72.19,73.58]	77.07**	[76.21,77.91]
Sample size (unweighted)	271,677		60,202	

Note: Percentages adjusted for survey design. *p-value < 0.05 **p-value < 0.001 for change between 2008 and 2013

^a^Measured by a five-point Likert scale going from excellent to very poor and recoded into a dummy variable with ‶1″ representing excellent or good and ‶0″ representing fair, poor and very poor

^b^Chronic diseases included arthritis, cancer, diabetes, bronchitis/asthma, hypertension, heart disease, kidney failure, and depression

^c^Hospitalization rate excluded hospitalization due to birth of a child and adjusted for age structure using standard population from 2010

**Fig. 1:**
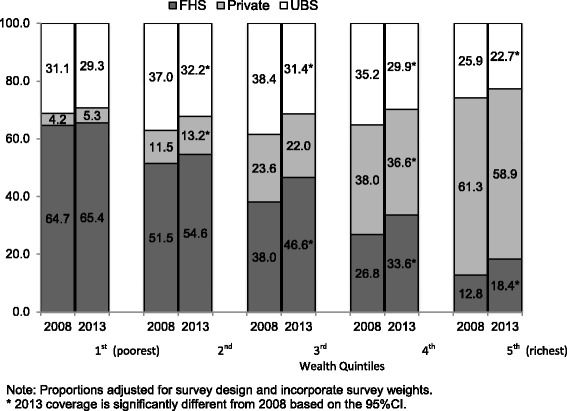
Type of healthcare coverage by quintiles of wealth index, Brazil, 2008 and 2013

Figure 1 shows the type of healthcare coverage in each quintile of the wealth index, by year. In the poorest quintile, enrollment in the FHS was reported by approximately 65 % of the respondents, and FHS coverage decreased with increasing wealth. People in the richest quintile reported the highest private plan coverage, approximately 60 %, and private coverage decreased with decreasing wealth, reaching only 5 % among the poorest in 2013. Between 2008 and 2013, FHS coverage increased significantly only in the top three quintiles, and private plan coverage appeared to be declining in the top three quintiles, but the change was only significant in the 4th quintile. The portion of people in the UBS category declined significantly between 2008 and 2013 among all quintiles, except the poorest.

Table 2 shows the adjusted prevalence ratios for predictors of each outcome. A doctor visit in the past year was higher in 2013, among females, older age groups, and people living in urban areas and richer regions of Brazil (Southeast and South), although people living in the Northeast also presented a significantly higher utilization of a doctor visit when compared to the North region. Having had a doctor visit was also higher among those with higher education, those enrolled in the FHS (*PR* = 1.05, *p* < 0.001) or a private plan (*PR* = 1.34, *p* < 0.001), and those with moderate (*PR* = 1.31, *p* < 0.001) and high health needs (and *PR* = 1.56, *p* < 0.001), but was lower among the poor versus the non-poor. The interaction between healthcare coverage and health needs was significant, and the marginal predicted probabilities varied from approximately 55 % among people with low needs who were enrolled in the FHS or UBS users, to 90 % among those with high needs regardless of the type of coverage, after accounting for all other predictors (Additional file 2). The interaction between being poor and type of coverage was significant and positive, which indicated that being enrolled in the FHS or a private plan was associated with a narrower gap between the poor and non-poor, as compared to UBS users (Additional file 3). All prevalence ratios were adjusted for sex, age, race, place of residence, literacy, formal education, type of healthcare coverage, wealth (poor vs. not poor), and health needs.

**Table 2:** Predictors of health care utilization, Brazil

	Doctor visit	Dentist	Hospital admission	Usual Source of Care
2013 (v. 2008)	1.03***	1.11***	0.85***	1.05***
	1.02,1.04	1.08,1.13	0.80,0.91	1.03,1.06
Female (v. male)	1.25***	1.16***	1.04	1.07***
	1.24,1.26	1.14,1.19	0.98,1.11	1.06,1.08
Age 24–35 (v. 18–24)	1.06***	0.96**	1.22**	1
	1.04,1.08	0.93,0.99	1.08,1.38	0.98,1.01
Age 35–44	1.04***	0.9***	1.2**	1.01
	1.02,1.07	0.88,0.93	1.05,1.36	0.99,1.02
Age 45–54	1.05***	0.83***	1.12	1
	1.03,1.08	0.80,0.86	0.98,1.27	0.99,1.02
Age 55–64	1.07***	0.72***	1.12	1.01
	1.04,1.09	0.69,0.75	0.98,1.29	0.99,1.03
Age 65 and older	1.12***	0.55***	1.42***	1
	1.09,1.15	0.52,0.58	1.24,1.63	0.98,1.02
Non-white (v. white)	1	0.94***	1	0.99
	0.99,1.01	0.92,0.96	0.93,1.07	0.98,1.00
Live in rural (v. urban)	0.98*	1	0.91*	0.99
	0.96,1.00	0.96,1.04	0.83,0.99	0.97,1.02
Northeast Region (v. North)	1.02*	1.12***	0.81***	0.99
	1.00,1.04	1.07,1.17	0.73,0.90	0.96,1.02
Southeast	1.08***	1.1***	0.81***	1.11***
	1.06,1.11	1.06,1.15	0.72,0.91	1.08,1.14
South	1.08***	1.21***	1.03	1.13***
	1.05,1.10	1.16,1.26	0.92,1.15	1.10,1.17
Center	1.04***	1.11***	1.09	1.01
	1.02,1.06	1.06,1.16	0.98,1.21	0.98,1.05
Literate (v. iliterate)	1.03***	1.59***	0.82***	0.99
	1.02,1.05	1.49,1.69	0.75,0.90	0.98,1.01
Primary complete (v. less than primary)	1.03***	1.23***	0.96	0.99
	1.02,1.05	1.19,1.27	0.88,1.05	0.97,1.00
High school complete	1.07***	1.4***	0.93	0.98**
	1.05,1.08	1.36,1.44	0.85,1.02	0.97,0.99
College or more	1.11***	1.63***	0.95	0.99
	1.09,1.12	1.58,1.69	0.83,1.07	0.97,1.01
FHS (v. UBS)	1.05***	1.04*	1.22*	1.14***
	1.02,1.08	1.00,1.07	1.02,1.46	1.11,1.17
Private^a^	1.34***	1.23***	2.07***	1.14***
	1.31,1.37	1.20,1.27	1.73,2.46	1.11,1.16
Poor (v. not poor) ^b^	0.93***	0.76***	1.08	1.01
	0.91,0.95	0.73,0.79	0.96,1.22	0.99,1.03
Moderate need (v. low) ^c^	1.31***	1.04***	3.15***	1.08***
	1.28,1.35	1.02,1.07	2.69,3.69	1.06,1.10
High need^c^	1.56***	0.98	5.97***	1.15***
	1.53,1.59	0.95,1.01	5.18,6.87	1.12,1.17
FHS#Poor	1.03*	1.11***	1.08	1.04**
	1.00,1.05	1.05,1.17	0.93,1.25	1.01,1.06
Private Plan#Poor	1.04**	1.12***	0.78**	0.99
	1.02,1.07	1.05,1.19	0.65,0.93	0.96,1.02
FHS#Moderate need	1	−	0.81*	0.95***
	0.97,1.03		0.65,0.99	0.92,0.97
FHS#High need	0.98	−	0.83	0.92***
	0.95,1.01		0.69,1.00	0.89,0.94
Private Plan#Moderate need	0.86***	−	0.78*	0.97*
	0.84,0.88		0.63,0.97	0.94,0.99
Private plan#High need	0.76***	−	0.71***	0.91***
	0.74,0.78		0.58,0.86	0.88,0.93
N	317462	317462	317462	317462

Note: Results are prevalence ratios and 95 % CIs from multivariable Poisson regression models that included all covariates and controlled for survey design. * *p* < 0.05; ** *p* < 0.01; *** *p* < 0.001. Covariates included were sex, age, race, place of residence, literacy, formal education, type of healthcare coverage, wealth (poor vs. not poor), and health need

^a^Private health insurance that only covers dental care was included in the category “private plan” only for the outcome probability of dentist visit

^b^Being poor was defined as being at the bottom 40 % (two lowest quintiles) of the distribution of the wealth index

^c^Health needs were estimated by applying factor analysis to the variables self-reported health status, reporting of activities interrupted due to health problems, reporting of chronic diseases, and reporting of comorbidity. The latent variable was then categorized into low need (bottom 50 %), high need (top 25 %) and moderate need (25 % between low and high)

Having a dental visit in the past year was significantly higher in 2013 than in 2008, higher among females, younger age groups, people living in regions other than the North, among literate people, those with more education (*PR* = 1.63, *p* < 0.001, for respondents with college education), and those with a private plan (*PR* = 1.23, *p* < 0.001) or enrolled in the FHS (*PR* = 1.04, *p* < 0.05) when compared to UBS users. Poorer people had a lower utilization of dentist visits, and the interaction between being poor and coverage was significant and positive, indicating that enrollment in FHS or a private plan was associated with narrower gaps between the rich and poor (Additional file 3).

Hospital admission was 15 % lower overall in 2013 than in 2008 (*PR* = 0.85, *p* < 0.001). Reporting of hospitalization in the past year was higher among respondents aged 24 to 44 years and those 65 and older, among people enrolled in the FHS (*PR* = 1.22, *p* < 0.05) or a private plan (*PR* = 2.07, *p* < 0.001), and among those with moderate (*PR* = 3.15, *p* < 0.001) and high health needs (*PR* = 5.97, *p* < 0.001), but was lower among people living in rural areas, residents of Northeast and Southeast regions, and the literate. The interaction between health needs and coverage type was significant and the predicted probability of hospitalization ranged from 1.9 % (UBS users) to 3.8 % (private plan) for those with low needs, and from 13 % (UBS) to 17 % (private plan) for those with high health needs (Additional file 2). The interaction between being poor and coverage type was significant and negative only for users of private plan. The probabilities of hospitalization indicated that among those with a private plan, poor and non-poor were hospitalized at similar probabilities (8.1 % vs. 7.5 %), while among the users of FHS and UBS the gap between poor and non-poor was relatively wider (for example, 7.1 % vs. 5.1 % for FHS) (Additional file 3).

The adjusted prevalence of a usual source of care (USC) was higher in 2013 and among females, those living in the richest regions of the country (Southeast and South), those with moderate and high health needs, and those with FHS or private plan coverage (*PR* = 1.14, *p* < 0.001 in both coverage types), but it was lower among those with high school education complete compared to those with less than primary education. The interaction between health needs and healthcare coverage was significant and marginal probabilities of having a USC varied from 65 % among people with low needs who were users of UBS to around 80 % among those with high needs enrolled in either FHS or a private plan (Additional file 2). The interaction between poor and coverage type was significant and positive only for FHS users: the probability of having a USC was the highest among the poor users of the FHS (80 %), but it was also high among users of private health plans poor and non-poor (Additional file 3).

Table 3 shows the Horizontal Equity Index (HEI) and the adjusted HEI for all the outcomes and years. Having a doctor visit was more concentrated among the rich (pro-rich distribution) in both years. Pro-rich inequity in the utilization of doctor’s visits did not change significantly between 2008 and 2013, but it increased significantly as a percentage of the feasible upper limit of the inequity (22.7 % of the total inequity possible in 2013 versus only 17.8 % in 2008). The distribution of dental visits was even more concentrated among the rich than the distribution of doctor’s visits, but it did not change significantly between 2008 and 2013. Inequity in hospitalization was pro-rich in 2008, but it was no different from zero in 2013, meaning that, hospitalizations were distributed equally across individuals from different socioeconomic backgrounds, after accounting for need. This pattern was observed using the unadjusted and adjusted HEI. Finally, reporting of a USC was more concentrated among the rich but the level of inequity was very small (prevalence-adjusted HEI was 3.5 % in 2008) with no significant change between 2008 and 2013.

**Table 3:** Horizontal inequity in healthcare utilization, Brazil, 2008 and 2013

	Health Equity Index (HEI) (95 % CI)		Prevalence-adjusted^a^ HEI (95 % CI)	
	2008	2013	2008	2013
Doctor visit	0.0537	0.0586	0.178	0.227
	﻿(0.051;0.056)	﻿(0.054;0.063)	﻿(0.169;0.185)	﻿(0.209;0.244)
Dentist visit	0.1715	0.1649	0.281	0.297
	﻿(0.165;0.178)	﻿(0.155;0.175)	﻿(0.270;0.292)	﻿(0.279;0.315)
Hospitalization	0.0321	0.0195	0.034	0.021
	﻿(0.02;0.045)	﻿(−0.011;0.05)	﻿(0.021;0.048)	﻿(−0.012;0.053)
Usual Source of Care	0.0096	0.0085	0.035	0.037
	﻿(0.005;0.014)	﻿(0.003;0.014)	﻿(0.018;0.052)	﻿(0.013;0.061)

^a^ The prevalence-adjusted HEI takes into account the prevalence of the outcome to re-calculate the bounds of the HEI

Note: For both measures, positive values represent pro-rich distribution and negative values represent pro-poor distribution. The absolute value of the prevalence-adjusted HEI represents the proportion of inequity in relation to the feasible maximum HEI (upper bound of the index)

**Fig. 2:**
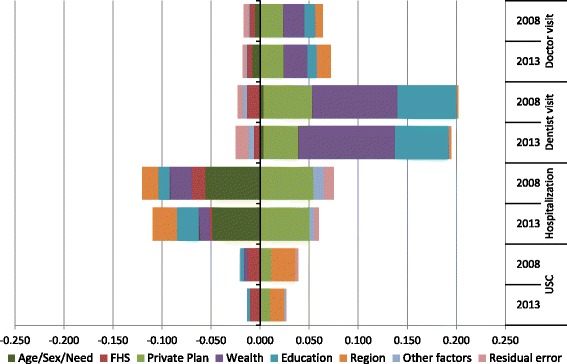
Contributions to the HEI from different predictors, Brazil, 2008 and 2013

Figure 2 presents the decomposition of components of the HEI for each outcome and year. Need predictors (age, sex and health needs) had a pro-poor contribution for doctor visits and hospitalization, meaning that health needs were positively associated with higher utilization and were also more concentrated among the poorest (negative contribution). Needs had a very small contribution for dentist visit and USC.

Enrollment in the FHS contributed to pro-poor distribution of all outcomes, but private health plans contributed to their pro-rich distribution. Wealth and education contributed to pro-rich inequity in doctor and dentist visits -- meaning that these predictors were positively associated with higher utilization and were also more concentrated among the richest in the social ranking -- but they contributed to pro-poor distribution of hospitalization. Region of residence had a similar pattern of pro-rich/pro-poor contribution as did wealth and education, but with a much smaller pro-rich contribution to dentist visit. Region of residence also contributed to pro-rich inequity in having a USC. Other predictors such as race and rural residence had very small contributions.

Between 2008 and 2013 the contribution of pro-rich and pro-poor were fairly consistent for all outcomes. However, for doctor visits, the total pro-rich contribution increased. For hospitalizations, the total pro-rich and pro-poor contribution declined.

## Discussion

Between 2008 and 2013, utilization of health care continued to increase among Brazil’s population, such that there were significant increases in the portion of the population reporting that they had seen a doctor and a dentist and had a usual source of care, while hospitalizations declined. These findings support the claim that Brazil’s population continued to see absolute gains in access to care despite recent economic crises. At the same time, our findings reveal that reductions in pro-rich inequity stalled for dentist visits and use of a USC while pro-rich inequity increased for utilization of doctor visits.

Our findings regarding pro-rich inequity was not particularly surprising given the uneven utilization patterns across different socioeconomic groups. The prevalence of seeing a doctor, dentist or having a USC was higher among those with more education, wealth, those living in the richest regions of the country, and those with private healthcare coverage -- similar to the situation observed in most OECD countries [37–39]. The findings regarding the pro-rich orientation of these predictors were also broadly consistent with the findings from OECD countries and previous studies in Brazil [20, 40, 41].

Health care inequity in Brazil seems to be higher than the inequity found in a sample of OECD countries but lower than other Latin American countries. In a group of 18 OECD countries, the prevalence-adjusted HEI for doctor visits varied from zero in the U.K. to 0.14 in Canada and 0.20 in the U.S.; for dentist visits the HEI varied from 0.07 in Switzerland to 0.15 in the U.K. and 0.3 in Canada and the U.S. (data from 2008 and 2009) [42]. On the other hand, unadjusted HEI for doctor visits and hospitalizations were respectively 0.079 and 0.015 in Chile [43] and 0.091and 0.036 in Colombia [44] (data from 2008 and 2009). However, comparing our results with those from other countries is problematic due to the different measures of socioeconomic position used; for instance, the studies on OECD countries and Chile used household income and the study on Colombia used household expenditure.

Concerning the changes in inequity in Brazil, our results suggest that the most recent period of the country’s history contrasts with the decade that preceded it when substantive reductions in inequity in health care were observed [20]. It may be that economic hardship prevented continued improvement towards greater equity, or that further reductions in inequity beyond the current level may be considerably more difficult to achieve. This is an area for further investigation.

At the same time, the pattern of inequity for each of the outcomes was not identical. For instance, increased inequity in doctor utilization seems to be linked to increased pro-rich orientation of the regional component. This finding is consistent with greater increases in FHS coverage among higher wealth quintiles and reflects regional patterns as some regions (Southeast and South) concentrate a larger portion of people in higher quintiles. The increase in utilization of a doctor visit coupled with a significant increase in prevalence-adjusted HEI from 2008 to 2013 could be an indication that as prevalence increased, those without access to doctor visits represent the poorest and hardest to reach with the programs implemented to date.

Despite increased pro-rich inequity in doctor visits, pro-rich inequity in having a usual source of care was small over the period studied. This pattern could be the result of barriers to doctor utilization among people who were able to identify a healthcare facility as a USC. For instance, doctor visits could be available during a limited number of hours each day or only a few days of the week, or the facility could be operating without a doctor on staff for a number of weeks due to the low contingent of doctors in some regions of the country.

Dental visits were the most pro-rich outcome and inequity declined considerably during the 1998–2008 period [19, 20]. Access to dental care has continuously increased between 2008 and 2013 [45] but further declines in inequity were not seen in the present study. Unlike doctor visits, disparities in dentist visits seem to be more strongly related to socioeconomic predictors (e.g. wealth, education) than regional differences.

Equity in the distribution of hospitalizations can be seen as an indicator of equal access to the most complex and expensive types of health care. Nevertheless, our findings indicate that hospitalization was strongly associated with private plan coverage, which could point to two non-excluding scenarios: one characterized by an overutilization of hospitalizations by those with a private plan, and the other characterized by poor access to elective procedures requiring hospitalization among the poorest without private coverage. The change from pro-rich inequity to equitable access may be an indicative of better use of hospitalization among people with a private plan, better access among the poorest, or both.

In terms of the three types of healthcare coverage, we found that being enrolled in either FHS or a private plan seems to have significantly narrowed the gap in utilization of doctor and dentist visits between the rich and poor, compared to UBS users. Poor people covered by the FHS had higher probability of having a USC, which is expected given the community-based nature of the FHS model.

In addition, the probability of a doctor visits and USC was high among those with high needs, independent of the type of coverage. This seems to indicate that public coverage is providing similar access to those with high needs as are private health plans. On the other hand, this might have more to do with the health seeking behavior of users with high needs than with access by type of coverage, as health needs are essential drivers of health care utilization. Health need was not an important predictor of dentist visits, probably because our need variables did not measure need for dental care. Variables measuring dental health needs are available in the PNS 2013 but not in the PNAD 2008. Further studies on dental care utilization should include such variables.

The strengths of this study include the use of a comprehensive set of outcomes and different analytical approaches that point to consistent patterns of service utilization. However, the study has some limitations. First, the PNAD and PNS had different designs, with the PNAD asking questions of all household members, which were answered by a proxy resident, while the PNS selected only one member to answer a more comprehensive questionnaire. In addition, the PNS was designed to produce more precise estimates [28]. These factors led to a PNS 2013 that is more robust and less susceptible to information bias than the PNAD. Nonetheless, this issue should not invalidate our conclusions given that data from both surveys showed expected patterns of sociodemographic and epidemiologic characteristics. Second, there is possible heterogeneity of perceived need among rich and poor. Even though many predictors of health care demand were included in our models, unmeasured factors may still drive some of these differences [46]. Third, the analysis was not able to differentiate between the actual type (private or public) of services used. We assume that those with a private health plan will use the private sector, but we do not know the extent to which users of the SUS may pay for private services out-of-pocket.

Finally, in contrast to studies examining health equity in the period 1998–2008, this study employed a wealth index to measure socioeconomic position. Sensitivity analyses using income data from the PNAD 2008 indicated that HEIs estimated from ranking individuals based on the wealth index were slightly larger than income-based HEIs (results not shown), which could explain the difference in values of HEI between this study and previous studies in Brazil [19, 20]. This issue, however, does not invalidate our findings regarding changes in inequity between 2008 and 2013. Also, sensitivity analyses using an alternative wealth index (constructed after randomly deleting some variables included in the PCA) provided generally robust results. 

## Conclusions

This study has shown that inequity in health care utilization in Brazil remains generally pro-rich and that the decline in pro-rich inequity observed in previous periods was not maintained in the period 2008–2013. While this study does not allow for an interpretation of the results in terms of the effect of the economic crisis, the finding could be an indicative of stagnant inequity levels for some outcomes, and in the case of doctor visits, a sign of deepening regional inequities. The pattern of increased disparities in doctor visits could be related to the latest phase of FHS expansion, with increasing coverage among people in the top quintiles of the wealth distribution, which generally live in the richest regions of the country. The decline in pro-rich inequity in hospitalizations, which became neutral in 2013, could be an indicative of better use of these services and higher access to complex procedures among the poorest. Considering the effect of the international economic crisis on government spending, it is important to understand that Brazil did not start implementing austerity measures by the time the study was fielded in 2013, but consequently there have been major cuts to many public social services including health care, which could further impact the distribution of services. The extent to which Brazil can continue investing in health care given the deepening financial crisis is an open question, but one that may have an impact on what has been, to date, a trend towards greater equity in essential health services within an otherwise highly unequal nation.

## Additional files

Additional file 1: Sample characteristics using individual datasets (PNAD 2008 and PNS 2013) vs. combined dataset. (DOCX 139 kb)
Additional file 2: Predicted probability of three outcomes showing the interaction between health need and type of coverage. (DOCX 24 kb)
Additional file 3: Predicted probability of four outcomes showing the interaction between a poverty indicator and type of coverage. (DOCX 27 kb)

## Abbreviations

FHS: Family health strategy
HEI: Horizontal equity index
PCA: Principal component analysis
PNAD: Pesquisa Nacional por Amostra de Domicílios
PNS: Pesquisa Nacional de Saúde
SUS: Sistema Único de Saúde
UBS: Unidade Básica de Saúde
USC: Usual source of care
