# Supplementary material for: Changes in health care inequity in Brazil between 2008 and 2013
Source: Int J Equity Health. 2016 Nov 17;15:140. doi: 10.1186/s12939-016-0431-8 (PMC5112635; doi:10.1186/s12939-016-0431-8)
Supplement: Additional file 1: — Sample characteristics using individual datasets (PNAD 2008 and PNS 2013) vs. combined dataset. (DOCX 139 kb) [file 12939_2016_431_MOESM1_ESM.docx]

Additional file 1: Sample characteristics using individual datasets (PNAD 2008 and PNS 2013) vs. combined dataset.

|  | Individual datasets | |  | Combined dataset | | |
| --- | --- | --- | --- | --- | --- | --- |
|  | PNAD - 2008 | PNS - 2013 |  | 2008 | 2013 | Total |
| Sex | | |  |  |  |  |
| female | 52.32 | 52.9 |  | 52.32 | 52.9 | 52.62 |
| CI | [52.14,52.50] | [52.15,53.64] |  | [52.15,52.49] | [52.12,53.67] | [52.21,53.04] |
| male | 47.68 | 47.1 |  | 47.68 | 47.1 | 47.38 |
| CI | [47.50,47.86] | [46.36,47.85] |  | [47.51,47.85] | [46.33,47.88] | [46.96,47.79] |
| Age groups | | |  |  |  |  |
| Age18_24 | 17.51 | 15.93 |  | 17.51 | 15.93 | 16.68 |
| CI | [17.31,17.71] | [15.36,16.51] |  | [17.31,17.71] | [15.35,16.53] | [16.36,17.01] |
| Age25_34 | 23.23 | 21.63 |  | 23.23 | 21.63 | 22.39 |
| CI | [22.98,23.48] | [21.04,22.22] |  | [23.00,23.47] | [21.02,22.24] | [22.05,22.73] |
| Age35_44 | 20.33 | 19.19 |  | 20.33 | 19.19 | 19.73 |
| CI | [20.11,20.55] | [18.64,19.76] |  | [20.12,20.54] | [18.62,19.78] | [19.41,20.06] |
| Age45_54 | 16.87 | 17.5 |  | 16.87 | 17.5 | 17.2 |
| CI | [16.67,17.07] | [16.96,18.05] |  | [16.67,17.07] | [16.94,18.07] | [16.89,17.51] |
| Age55_64 | 11.11 | 13.46 |  | 11.11 | 13.46 | 12.34 |
| CI | [10.93,11.29] | [12.97,13.97] |  | [10.94,11.28] | [12.96,13.98] | [12.06,12.63] |
| Age65andmore | 10.95 | 12.29 |  | 10.95 | 12.29 | 11.66 |
| CI | [10.75,11.15] | [11.79,12.82] |  | [10.75,11.15] | [11.77,12.84] | [11.36,11.95] |
| Race | | |  |  |  |  |
| white | 49.98 | 47.46 |  | 49.98 | 47.46 | 48.66 |
| CI | [49.38,50.58] | [46.66,48.27] |  | [49.14,50.83] | [46.44,48.49] | [48.01,49.31] |
| Black | 7.52 | 9.2 |  | 7.52 | 9.2 | 8.4 |
| CI | [7.28,7.77] | [8.74,9.67] |  | [7.26,7.80] | [8.70,9.72] | [8.11,8.70] |
| Asian | 0.66 | 0.94 |  | 0.66 | 0.94 | 0.81 |
| CI | [0.59,0.74] | [0.81,1.09] |  | [0.60,0.74] | [0.81,1.09] | [0.73,0.89] |
| Pardo | 41.53 | 41.98 |  | 41.53 | 41.98 | 41.77 |
| CI | [40.95,42.12] | [41.20,42.77] |  | [40.70,42.37] | [41.04,42.93] | [41.15,42.39] |
| indigenous | 0.3 | 0.42 |  | 0.3 | 0.42 | 0.36 |
| CI | [0.25,0.35] | [0.36,0.50] |  | [0.26,0.35] | [0.35,0.51] | [0.32,0.41] |
| Residence | | |  |  |  |  |
| rural | 15.37 | 13.79 |  | 15.37 | 13.79 | 14.54 |
| CI | [14.39,16.40] | [13.34,14.25] |  | [14.24,16.57] | [12.85,14.79] | [13.84,15.27] |
| Region | | |  |  |  |  |
| North | 7.35 | 7.44 |  | 7.35 | 7.44 | 7.4 |
| CI | [6.87,7.86] | [7.25,7.64] |  | [6.43,8.39] | [7.01,7.89] | [6.92,7.90] |
| Northeast | 26.42 | 26.62 |  | 26.42 | 26.62 | 26.52 |
| CI | [25.98,26.86] | [26.11,27.14] |  | [24.67,28.24] | [25.42,27.85] | [25.55,27.52] |
| Southeast | 44.14 | 43.79 |  | 44.14 | 43.79 | 43.96 |
| CI | [43.62,44.66] | [43.14,44.45] |  | [42.69,45.60] | [42.32,45.28] | [42.90,45.03] |
| South | 14.82 | 14.78 |  | 14.82 | 14.78 | 14.8 |
| CI | [14.47,15.17] | [14.37,15.20] |  | [14.29,15.36] | [13.89,15.72] | [14.25,15.37] |
| Center | 7.27 | 7.36 |  | 7.27 | 7.36 | 7.32 |
| CI | [7.08,7.46] | [7.17,7.57] |  | [7.00,7.55] | [6.96,7.79] | [7.08,7.57] |
| Iliterate | | |  |  |  |  |
| yes | 89.37 | 91.52 |  | 89.37 | 91.52 | 90.5 |
| CI | [89.05,89.69] | [91.11,91.91] |  | [88.86,89.87] | [91.05,91.97] | [90.16,90.83] |
| Education level | | |  |  |  |  |
| less_primary | 45.88 | 38.93 |  | 45.88 | 38.93 | 42.24 |
| CI | [45.31,46.44] | [38.08,39.80] |  | [45.13,46.63] | [37.98,39.89] | [41.61,42.87] |
| primary_complete | 16.42 | 15.53 |  | 16.42 | 15.53 | 15.95 |
| CI | [16.18,16.66] | [14.99,16.08] |  | [16.18,16.66] | [14.97,16.10] | [15.64,16.27] |
| HS_complete | 29.01 | 32.8 |  | 29.01 | 32.8 | 31 |
| CI | [28.61,29.42] | [32.10,33.52] |  | [28.54,29.49] | [32.03,33.59] | [30.53,31.48] |
| college_more | 8.69 | 12.74 |  | 8.69 | 12.74 | 10.81 |
| CI | [8.38,9.01] | [12.04,13.47] |  | [8.36,9.03] | [11.99,13.52] | [10.39,11.25] |
| Self-rated health |  |  |  |  |  |  |
| Fair/poor | 28.7 | 32.24 |  | 28.7 | 32.24 | 30.55 |
| CI | [28.32,29.07] | [31.53,32.96] |  | [28.26,29.13] | [31.48,33.00] | [30.12,30.99] |
| Activity interrupted | |  |  |  |  |  |
| yes | 8.93 | 8.06 |  | 8.93 | 8.06 | 8.47 |
| CI | [8.72,9.14] | [7.67,8.47] |  | [8.73,9.14] | [7.66,8.48] | [8.24,8.71] |
| At least on chronic | |  |  |  |  |  |
| yes | 31.91 | 35.34 |  | 31.91 | 35.34 | 33.71 |
| CI | [31.59,32.24] | [34.65,36.04] |  | [31.59,32.24] | [34.60,36.08] | [33.29,34.13] |
| Two or more chronic | |  |  |  |  |  |
| yes | 12.22 | 12.71 |  | 12.22 | 12.71 | 12.47 |
| CI | [12.01,12.43] | [12.24,13.19] |  | [12.01,12.43] | [12.20,13.23] | [12.19,12.76] |
| FHS |  |  |  |  |  |  |
| yes | 48.79 | 54.61 |  | 48.79 | 54.61 | 54.96 |
| CI | [47.66,49.93] | [53.33,55.88] |  | [47.48,50.10] | [59.74,62.68] | [53.33,55.88] |
| med_plan |  |  |  |  |  |  |
| yes | 26.16 | 26.4 |  | 26.16 | 26.4 | 26.28 |
| CI | [25.61,26.71] | [25.53,27.28] |  | [25.44,26.89] | [25.44,27.37] | [25.69,26.89] |
| Doc_visit |  |  |  |  |  |  |
| yes | 69.81 | 74.2 |  | 69.81 | 74.2 | 72.12 |
| CI | [69.42,70.20] | [73.48,74.91] |  | [69.40,70.22] | [73.45,74.95] | [71.67,72.56] |
| Dentist visit |  |  |  |  |  |  |
| yes | 38.97 | 44.43 |  | 38.97 | 44.43 | 41.84 |
| CI | [38.51,39.45] | [43.63,45.23] |  | [38.47,39.49] | [43.56,45.31] | [41.31,42.37] |
| Hospitalization |  |  |  |  |  |  |
| yes | 6.659 | 5.725 |  | 6.659 | 5.725 | 6.161 |
| CI | [6.508,6.814] | [5.412,6.054] |  | [6.509,6.812] | [5.396,6.072] | [5.971,6.356] |
| USC |  |  |  |  |  |  |
| yes | 72.89 | 77.07 |  | 72.89 | 77.07 | 75.08 |
| CI | [72.15,73.61] | [76.27,77.86] |  | [72.14,73.62] | [76.18,77.94] | [74.50,75.66] |

Estimates adjusted for survey design.
