# Supplementary material for: Changes in health care inequity in Brazil between 2008 and 2013
Source: Int J Equity Health. 2016 Nov 17;15:140. doi: 10.1186/s12939-016-0431-8 (PMC5112635; doi:10.1186/s12939-016-0431-8)
Supplement: Additional file 3: — Predicted probability of four outcomes showing the interaction between a poverty indicator and type of coverage. (DOCX 27 kb) [file 12939_2016_431_MOESM3_ESM.docx]

Panel 2: Predicted probability of four outcomes representing interaction between a poverty indicator and type of coverage, Brazil (2008-2013). Top row (from left to right): doctor visit and dentist visit; Bottom row: hospitalization and USC.
